# Chemically stable inhibitors of 14-3-3 protein–protein interactions derived from BV02

**DOI:** 10.1080/14756366.2019.1574779

**Published:** 2019-02-07

**Authors:** Leire Iralde-Lorente, Ylenia Cau, Letizia Clementi, Lorenzo Franci, Giusy Tassone, Daniela Valensin, Mattia Mori, Adriano Angelucci, Mario Chiariello, Maurizio Botta

**Affiliations:** a Department of Biotechnology, Chemistry and Pharmacy, Università degli Studi di Siena, Siena, Italy;; b Department of Biotechnological and Applied Clinical Sciences, University of L’Aquila, L’Aquila, Italy;; c Istituto per lo Studio, la Prevenzione e la Rete Oncologica (ISPRO), Siena, Italy;; d Department of Medical Biotechnologies, PhD course GenOMeC, Università degli Studi di Siena, Siena, Italy;; e Consiglio Nazionale delle Ricerche, Istituto di Fisiologia Clinica, Siena, Italy;; f Center for Biotechnology, College of Science and Technology, Sbarro Institute for Cancer Research and Molecular Medicine, Temple University, Philadelphia, PA, USA;; g Lead Discovery Siena s.r.l., Siena, Italy

**Keywords:** 14-3-3, protein–protein interaction, c-Abl, leukaemia, 4-aminoantipyrine

## Abstract

14-3-3 are regulatory proteins that through protein–protein interactions (PPI) with numerous binding partners could be involved in several human diseases, including cancer, neurodegenerative disorders, and pathogens infections. Following our research interest in the development of 14-3-3 PPI inhibitors, here we exploited the privileged 4-aminoantipyrine scaffold in the design and synthesis of some derivatives endowed with antiproliferative activity against K-562 cells, and capable of binding to recombinant 14-3-3σ as evidenced by NMR spectroscopy. The binding mode was further explored by molecular modelling, while coupling confocal microscopy with intensitometric analysis showed that compound **1** was able to promote the nuclear translocation of c-Abl at low micromolar concentrations. Overall, **1** is chemically stable compared to parent 14-3-3 PPI inhibitors, and thus emerged as a confirmed hit for further development.

## Introduction

14-3-3 is a family of eukaryotic regulatory proteins that interact with a multitude of different targets by means of protein–protein interactions (PPI). To date, more than 500 partners of 14-3-3 have been identified through biochemical and bioinformatics studies, most of which are disease-relevant phosphorylated proteins sharing the optimal binding sequence R(X)XpS/TXP^1^. In recent years, 14-3-3 proteins have emerged as profitable targets in the therapy of several diseases including different types of cancer, neurodegenerative disorders, and pathogens infections^1–4^. In humans, seven different isoforms have been characterised, which are commonly named with Greek letters σ, ζ, β, γ, η, ε, and τ^5^^,^^6^. Although expression levels of specific 14-3-3 isoforms might vary in different tissues, organs, or in particular disease conditions, these isoforms share a significantly high degree of sequence and structural conservation^7^. Particularly, the binding site of phosphopeptides, which is located within the so-called amphipathic groove and is composed by a number of basic residues, is among the most conserved portion of 14-3-3.

**Figure 1.**
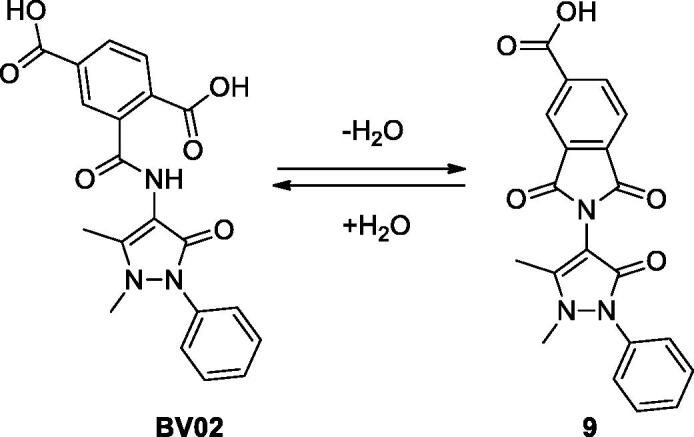
Chemical structure of the reference 14-3-3 PPI inhibitors **9** and **BV02**, which are connected through a hydration/dehydration pathway.

Our research group has long been involved in targeting 14-3-3 proteins as a strategy to treat chronic myeloid leukaemia (CML)^8–10^. The first non-peptidic inhibitor of 14-3-3/c-Abl interaction (namely, **BV02**, Figure 1) has been discovered by virtual screening of a commercial library of compounds. The molecule was found to promote c-Abl translocation into the nucleus and to provide antiproliferative effects also in CML cells expressing the Imatinib-resistant T315I Bcr-Abl construct^8^. Since then, **BV02** has been used as tool compound in multiple biological studies aimed at investigating the pharmacological inhibition of 14-3-3 PPI. Notwithstanding, the molecule suffers from chemical instability in aqueous buffer, which leads to the phthalimide derivative **9** by means of a hydration/dehydration pathway (Figure 1)^11^. Thus, the characterisation of the mechanism of action of **BV02** has required a combination of chemical, computational, and spectroscopic studies, which have led to the identification of the phthalimide **9** as the bioactive form of **BV02** in physiological conditions, although the persistency of **9** was found to be strictly dependent on the pH as well as the incubation time^12^.

To further shed light on the mechanism of 14-3-3 PPI inhibition by the privileged 4-aminoantipyrine scaffold, and to identify novel derivatives endowed with pharmacological properties comparable to **BV02**/**9** but endowed with a suitable chemical stability profile, here we designed and synthesised three derivatives of the 14-3-3 PPI inhibitor **9**. Interaction with 14-3-3σ was monitored by NMR spectroscopy and molecular modelling, while the mechanism of action and anticancer potential were assayed in *in vitro* cancer cell models.

## Results and discussion

### Chemistry

**Scheme 1.**
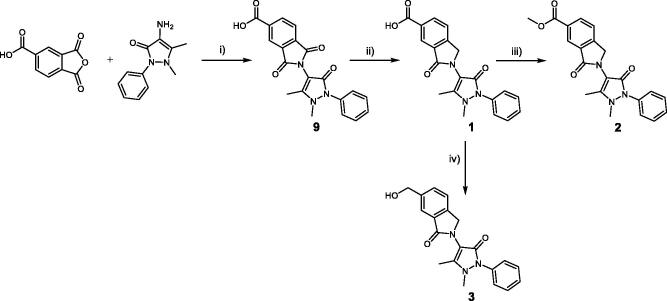
(i) DMAP, anhydrous DMF, r.t. for 72 h. (ii) Sn, HCl, AcOH, 80 °C for 24 h. (iii) (COCl)_2_, DMF, THF for 1 h, then MeOH at 0 °C. (iv) BH_3_·Me_2_S at 0 °C then stirred at r.t. for 12 h.

Compounds **1**–**3** were synthesised using the synthetic route outlined in Scheme 1. Starting from 4-aminoantipyrine and 1,2,4-benzenetricarboxylic anhydride, compound **9** was obtained in quantitative yield after stirring at room temperature (r.t.) for 72 h. Then, it was treated with AcOH and H_2_SO_4_ in the presence of tin powder, which selectively reduces the carbonyl group of the phthalimide moiety, to afford compound **1** in a high yield (79%). To afford compound **2**, acid chloride was prepared by reacting compound **1** with oxalyl chloride and subsequently methylated by the addition of MeOH into the reaction mixture. Synthesis of compound **3** was completed after the reduction of the carboxylic acid^1^ by borane dimethyl sulphide 1 M, leading to the primary alcohol **3**.

**Figure 2.**
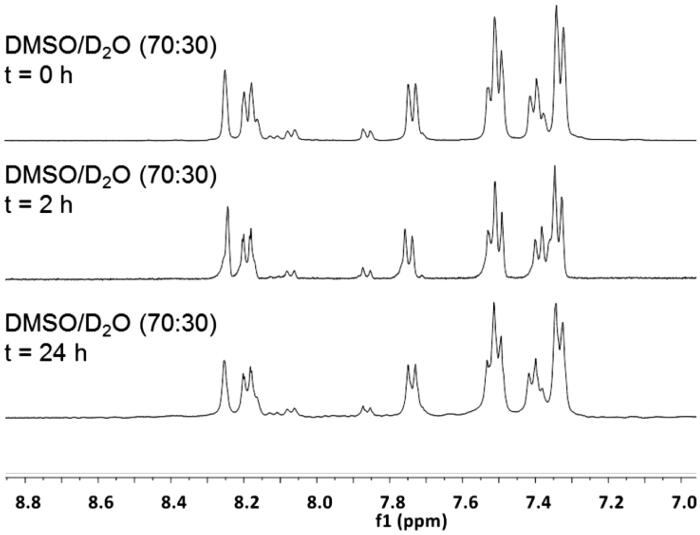
Aromatic region of ^1^H NMR spectra of molecule **1** in DMSO/D_2_O (70:30) at r.t. recorded and different time points.

Compared to the parent 14-3-3 PPI inhibitor **9**, chemical stability at physiological conditions was remarkably improved by removing one of the carbonyl groups from the phthalimide moiety (compound **1**), as observed by NMR spectroscopy (Figure 2). Indeed, no variations in peaks chemical shift and multiplicity within the aromatic region of the NMR spectrum of **1** in DMSO/D_2_O 70:30 was observed, also after 24 h. According to the previous analysis of the chemical stability of **9**^12^, this region was selected as the most sensible to the hydration/dehydration pathway shown in Figure 1. Besides NMR, chemical identity of compound **1** was confirmed also by mass spectrometry after 24 h.

### Antiproliferative activity of 1–3

**Figure 3.**
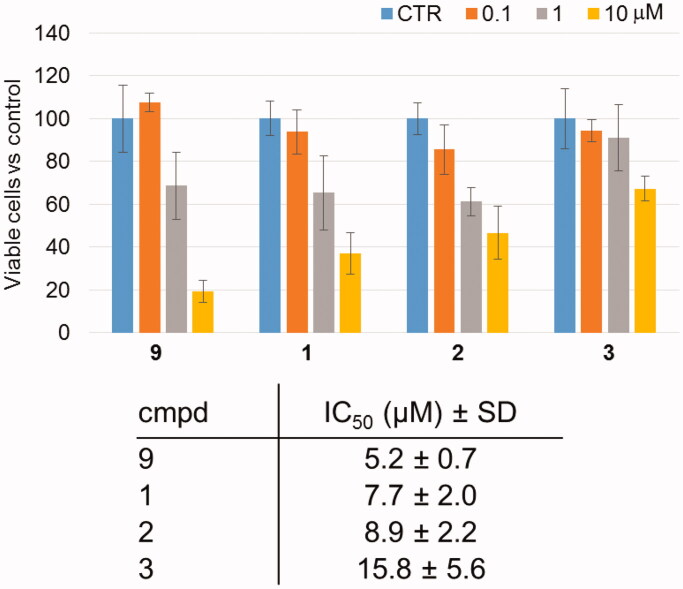
Antiproliferative activity of **1**–**3** and the parent compound **9** evaluated by *in vitro* analysis of percentage of viable K-562 cells with respect untreated cells (100%) after 72 h of incubation. Respective cytostatic IC_50_ values (±SD) are shown in the table. CTR = DMSO vehicle (<0.1%).

Compounds **1**–**3** and the reference 14-3-3 PPI inhibitor **9** were evaluated for their biological activity by *in vitro* model of human erythroleukemia. Cancer cells K-562 were matched with increasing concentration of the molecules, and incubated in standard culture conditions for 72 h. Control K-562 cells were cultured with a percentage of DMSO vehicle (<0.1%) corresponding to that present in the highest concentration of the compounds. At the end point, cell number and viability were evaluated by automatic cell counter, and mean number of viable cells was expressed as percentage with respect to control cells (Figure 3). Compounds **1** and **2** showed a significant reduction in viable cells with respect to the control starting from the concentration of 1 µM. Notably, their activity is highly comparable to that of the reference compound **9**. The calculation of the concentration able to reduce by 50% the number of viable cells with respect to control demonstrated that evaluated compounds have IC_50_ ranging from 5.2 to 15.8 µM (Figure 3). Compared to **1**, **2**, and **9**, compound **3** affects the proliferation of K-562 cells to a lesser extent. It is worth noting that these data are in agreement with rough structure-activity relationships (SAR) previously described for **BV02** and compound **9**^11^, showing that the carboxylic acid is a suitable functionality for the pharmacophore to provide antiproliferative activity through inhibition of 14-3-3 PPI, whereas the methyl ester is less effective. Following this trend, the primary alcohol **3** showed indeed the weakest antiproliferative activity.

### Predicted binding mode of 1–3 to 14-3-3σ

**Figure 4.**
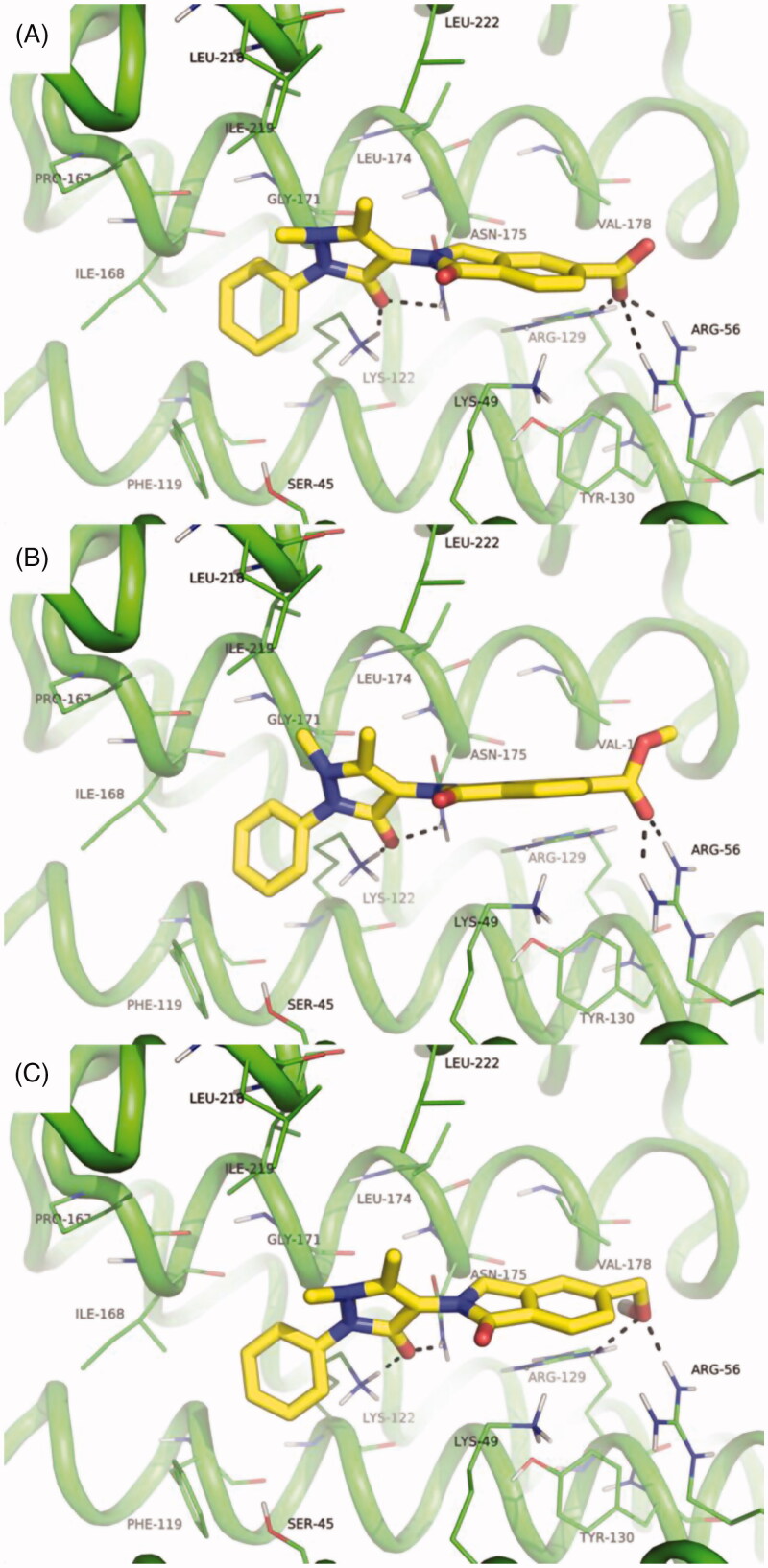
Predicted binding mode of compound **1** (A), **2** (B), and **3** (C) within the amphipathic groove of 14-3-3σ. The crystallographic structure of the protein coded by PDB ID: 1YWT is shown as green cartoon; residues within 4 Å from the ligands are shown as lines and are labeled. Ligand/protein polar contacts are highlighted by black dashed lines. Ligands are shown as yellow sticks.

The possible binding mode of compounds **1**–**3** to the crystallographic structure of 14-3-3σ was investigated by molecular docking simulations, which were carried out as described previously^11^. Results clearly show that all compounds are able to fit the amphipathic groove of 14-3-3, which is the well-known binding site of 14-3-3’s interaction partners. Moreover, the molecules interact with the side chain of Arg56 and Arg129 that are relevant for the binding of phosphorylated serine residues of 14-3-3 partners (Figure 4). The key residue Lys49 is implicated in cation-π interactions with the aromatic portion of the molecules. Additional interactions are established with the side chain of Asn175 and Lys122, while the hydrophobic portion of the 4-aminoantipyrine occupies a non-polar part of the amphipathic groove in proximity of Phe119, Ile168, Leu218, Ile219, and Leu222 (Figure 4).

It is worth mentioning that compounds **1**–**3** share a highly consistent binding mode, which is also comparable to that already described for the parent compound **9**, with only a small difference in the orientation of the N-methyl group of the pyrazolone ring. This is indeed a consequence of the possibility to flip pyramidal N atoms in GOLD docking program and does not affect significantly the overall binding mode of **1**–**3**.

### Compounds 1 and 2 bind to recombinant 14-3-3σ

The direct binding of **1** and **2** to recombinant 14-3-3σ was preliminarily assessed by screening techniques such as fluorescence polarisation (FP) and surface plasmon resonance (SPR) (data not shown). However, results were difficult to interpret, most likely because of the relatively weak affinity of the molecules for 14-3-3σ. Thus, we decided to monitor the interaction between recombinant 14-3-3σ and compounds **1** and **2** through ^1^H NMR spectroscopy by running transferred nuclear overhauser effect (Tr-NOE) experiments. This approach is well established and relies on the slow tumbling of the ligand when it is bound to specific protein partners. In fact, ligand–protein interaction greatly enhances the efficiency of the proton-proton NOE cross relaxation between protons of the ligand^13^.

**Figure 5.**
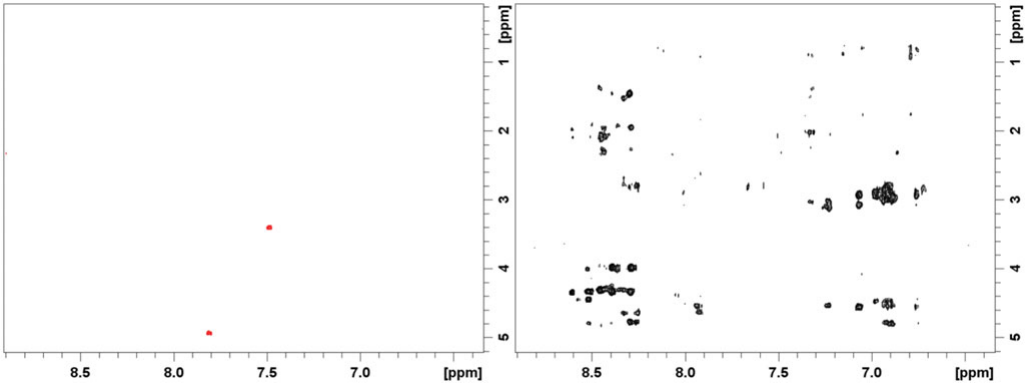
Selected region of ^1^H-^1^H NOESY spectra of compound **2** 0.5 mM (left) and 14-3-3σ 0.2 mM (right), at pH 7.0 and 298 K. Negative and positive peaks are shown as red and black contours.

Because of their size, **1** and **2** have negative NOE correlations (positive NOEs enhancements) while 14-3-3-σ has positive NOE correlations (negative NOE enhancements), as highlighted in Figure 5.

**Figure 6.**
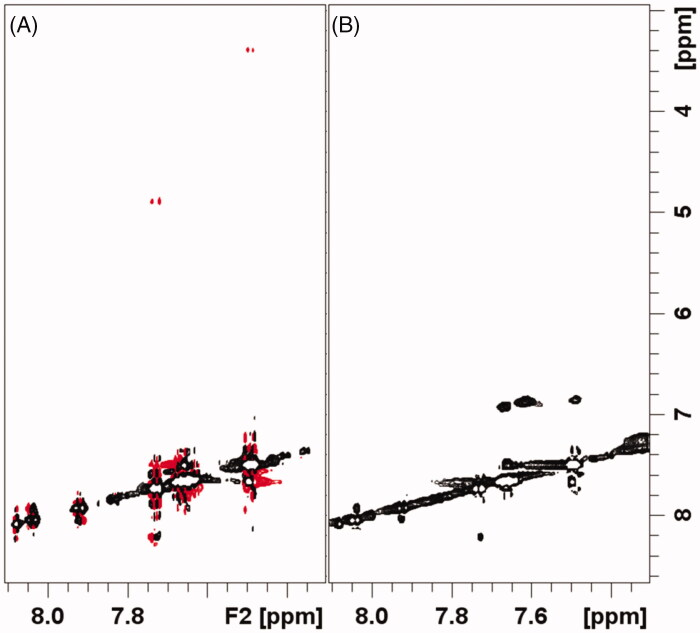
Selected regions of ^1^H-^1^H NOESY spectra of compound **1** alone 0.5 mM pH 7.0 (A), and compound **1** 0.5 mM + 0.2 eqs. of 14-3-3s pH 7.0 (B).

**Figure 7.**
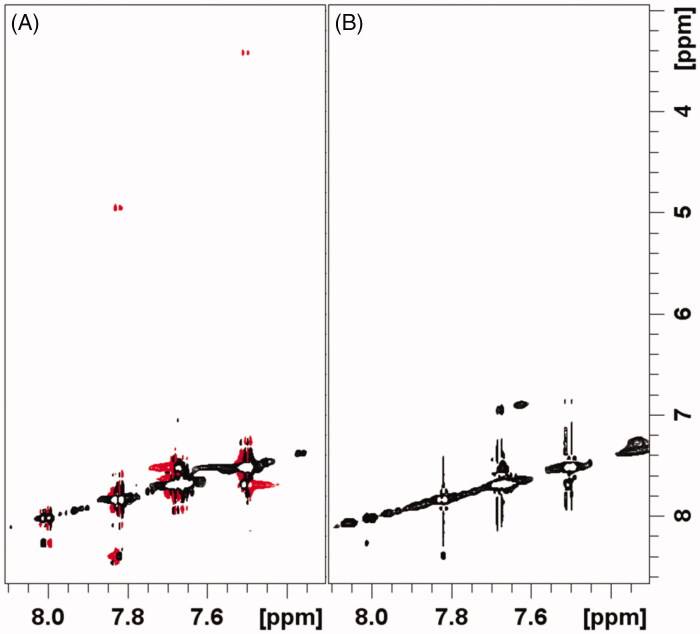
Selected regions of ^1^H-^1^H NOESY spectra of compound **2** alone 0.5 mM pH 7.0 (A), and compound **2** 0.5 mM + 0.2 eqs. of 14-3-3s pH 7.0 (B).

In the presence of the ligand–protein interaction, if the ligand off-rate is fast compared to the bound NOE cross relaxation rate, the Tr-NOESY signal intensities are the weighted average between the bound and free cross-peaks intensities^14^. As shown in Figures 6 and 7, the intensities of NOE cross-peaks of **1** and **2** changes from negative to zero, upon 14-3-3σ addition. This behaviour is consistent with the interaction between the two molecules and 14-3-3σ. The zero intensities of NOEs might be explained by considering the occurrence of positive NOE peaks due to the presence of small fractions of either **1** or **2** bound to 14-3-3σ.

The analysis of 1 D ^1^H spectra of either **1** or **2** does not reveal any significant variations after protein addition. In fact, the concentration of the protein-ligand adduct is too small to induce any perturbation in NMR signals. In addition, it is consistent with the results obtained by NOESY experiments that support the formation of complexes with fast ligand off‐rates and *K*_D_ values > 1 μM^15^^,^^16^.

### Compound 1 enhances the nuclear translocation of c-Abl

**Figure 8.**
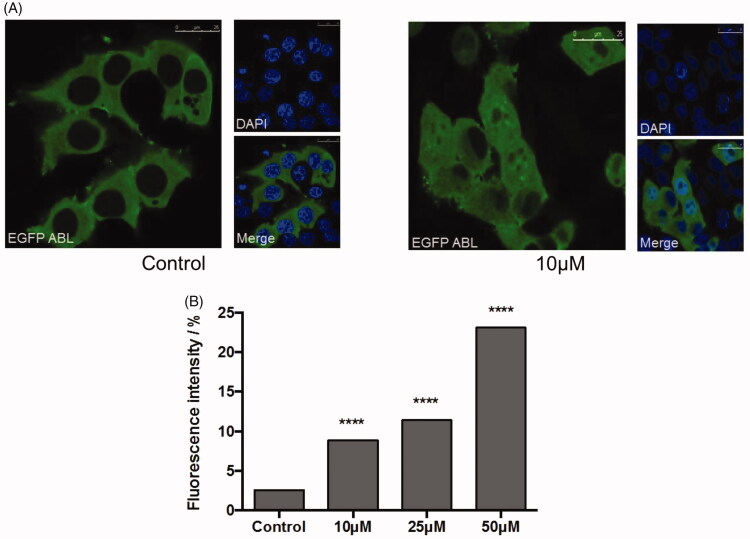
The 14-3-3 PPI inhibitor promotes nuclear translocation of c-Abl. (A) Confocal microscopy images showing subcellular localisation of c-Abl in HeLa EGFP-Abl cells untreated or treated with **1** at 10 μM for 24 h. (B) Intensitometric analysis of confocal microscopy images of nuclear EGFP fluorescence on HeLa EGFP-Abl cells treated with increasing concentrations of **1** for 24 h; bars represent the mean ± SEM of the nuclear EGFP fluorescence intensity of seven representative fields; ^****^p < .0001.

We have previously shown that the **BV02** inhibitor of 14-3-3/c-Abl interaction is able to promote c-Abl translocation into the nucleus and to provide antiproliferative effects also in CML cells expressing the Imatinib-resistant T315I Bcr-Abl construct^8^. We, therefore, next assessed nuclear translocation of c-Abl upon treatment with the most effective 14-3-3 PPI inhibitor **1**, using HeLa adenocarcinoma cells stably overexpressing EGFP-tagged c-Abl (HeLa EGFP-Abl) as a reporter system^11^. Confocal microscopy images of HeLa EGFP-Abl cells clearly show an increase in nuclear EGFP-Abl in the presence of **1**, compared to control (Figure 8(A)). Indeed, the effect of **1** on EGFP-Abl subcellular localisation, quantitatively scored by analysing the confocal microscopy images with the Volocity software (PerkinElmer Life Science, Waltham, MA) (Figure 8(B)), confirmed the ability of **1** to promote nuclear relocalisation of c-Abl in a dose-dependent manner. Hence, our molecule was able to promote strong c-Abl nuclear translocation, even in an experimental setting in which this protein is overexpressed, suggesting that similar doses of compound **1** might be largely effective on the c-Abl endogenous protein, ultimately explaining the antiproliferative effects observed in Bcr-Abl-expressing CML cells.

## Conclusion

Modulation of 14-3-3 proteins is a valuable tool to probe the regulatory functions of eukaryotic cells, and to develop effective therapeutic strategies against a number of human diseases. By exploiting the 4-aminoantipyrine scaffold of 14-3-3σ PPI inhibitors previously developed by our research group, here we designed and synthesised a number of derivatives endowed with enhanced chemical stability at physiological conditions. Particularly, compounds **1**–**3** showed antiproliferative effects against K-562 erythroleukemia cell line at low micromolar concentrations. Molecular modeling suggested a possible binding mode of these compounds that clearly emphasises the role of the carboxylic function. NMR spectroscopy further confirmed the direct binding of **1** and **2** to recombinant 14-3-3σ, while the most promising hit **1** was found to enhance the nuclear translocation of c-Abl in a dose-dependent manner by confocal microscopy and intensitometric analysis.

Overall, this study further confirms the relevance of the 4-aminoantipyrine scaffold in the inhibition of 14-3-3σ PPI, and highlights compound **1** as a valuable hit that is chemically stable at physiological conditions, and is suitable for further development.

## Materials and methods

### Chemistry

All commercially available chemicals and solvents were used as purchased. THF was dried over sodium and benzophenone prior to use. Anhydrous reactions were run under positive pressure of dry nitrogen. TLC was carried out using Merck TLC plates silica gel 60 F254 (Merck, Kenilworth, NJ). ^1^H NMR and ^13^C NMR were recorded at 400 and 100 MHz, respectively, on a Bruker AC200F spectrometer (Bruker, Billerica, MA). Proton shift for ^1^H NMR are reported in parts per million (δ scale) and internally referenced to the CDCl_3_ or DMSO signal, at 7.26 and 2.50 ppm, respectively. Chemical shifts for carbon are reported in parts per million (δ scale) and referenced to the carbon resonances of the solvent (CDCl_3_ at δ 77.16 and DMSO at δ 39.52 ppm). Data are shown as following: chemical shift, multiplicity (s = singlet, d = doublet, t = triplet, m = multiplet, and/or multiplet resonances), coupling constant (*J*) in Hertz (Hz), and integration. Mass spectra (MS) data were obtained using an Agilent 1100 LC/MSD VL system (G1946C) (Agilent Technologies, Palo Alto, CA) by direct injection with a 0.4 ml min^−1^ flow rate using a binary solvent system of MeOH:H_2_O (95:5). UV detection was monitored at 254 nm. MS were acquired in positive scanning mode over the mass range *m*/*z* 50–1500.

### Synthesis of the reference compound 9


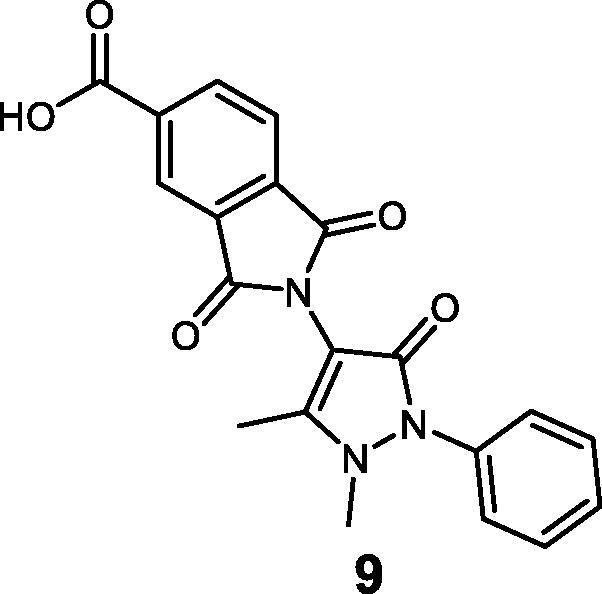


1,2,4-benzenetricarboxylic anhydride (1.06 g, 5.2 mmol, and 1 eq) and DMAP (190.58 mg, 1.56 mmol and 0.3 eq) were added to a solution of 4-aminoantipyrine (1 g, 5.2 mmol, and 1 eq) in DMF dry (15 ml), giving a yellow colour. The mixture was stirred at r.t. for 72 h. After this time, H_2_O (20 ml) was added and the solution was stirred 20 min until a white precipitate appeared. The white solid was filtered and washed with H_2_O and hexane.

**Yield:** quantitative

**^1^H NMR (400 MHz, DMSO, 298 K) (ppm):** 13.83 (s, OH); 8.43 (d*, J=* 7.8 Hz, 1H); 8.32 (s, 1H); 8.10 (d, *J=* 7.8 Hz, 1H); 7.55 (t, *J=* 7.8 Hz, 2H); 7.40 (m, 3H); 3.32 (s, 3H); 2.26 (s, 3H).**MS** (ESI) *m/z*: 377.8 [M + H]

### Synthesis compound 1


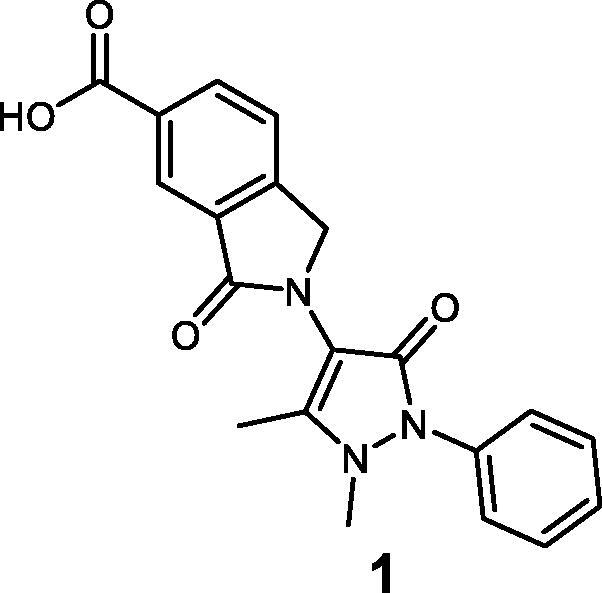


Compound **9** (120 mg, 0.318 mmol, and 1 eq) was dissolved in a mixture of concentrated HCl (5 ml) and glacial acetic acid (5 ml). Sn (226.5 mg, 1.908 mmol, and 6 eq) was added to the solution, and the mixture was stirred at 80 °C for 24 h before being cooled to r.t. and filtered through celite. The solution was extracted with AcOEt (30 ml × 3). The combined organic phases were washed with H_2_O and brine, and dried over anhydrous Na_2_SO_4_. The solvent was removed under vacuum to afford 92 mg of a white solid.

**Yield**: 79%

**^1^H NMR (400 MHz, DMSO, 298 K) (ppm):** 8.22 (m, 2H); 7.78 (m, 1H); 7.51 (m, 2H); 7.38 (m, 3H); 4.87 (s, 2H); 3.17 (s, 3H); 2.23 (s, 3H). **^13^C NMR (100 MHz, DMSO, 298 K) (ppm):** 167.13, 166.48, 161.55, 153.09, 147.40, 135.07, 133.13, 132.36, 131.39, 129.67, 127.25, 124.64, 124.36, 107.04, 51.09, 36.03, 11.67. **MS** (ESI) *m/z*: 363.8 [M + H], 385.8 [M + Na]

### Synthesis compound 2


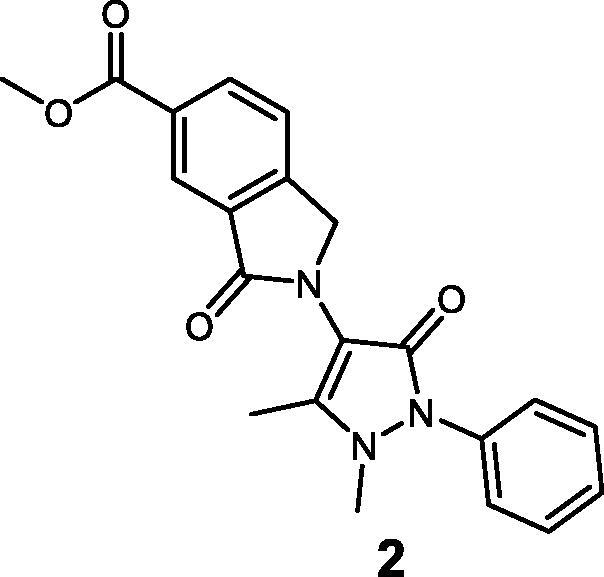


Oxalyl chloride (0.33 ml, 3.738 mmol, and 14 eq) was slowly added to a solution of compound **1** (100 mg, 0.267 mmol, and 1 eq) in THF (4 ml) at 0 °C. The mixture was then stirred for 15 min, and anhydrous DMF (0.1 ml) was added dropwise. The solution was stirred for 1 h at r.t. and then cooled again to 0 °C. MeOH was added to the reaction and left under stirring for 15 min. The reaction was quenched by the addition of NaOH 2 N, and then extracted with AcOEt (15 ml × 3). The organic layer was washed with NaHCO_3_, brine, dried over Na_2_SO_4_ and evaporated under reduced pressure to obtain 49 mg of a brown oil. The mixture was purified by column chromatography (DCM/MeOH 95:5) to give a yellow solid.

**Yield:** 47%

**^1^H NMR (400 MHz, DMSO, 298K) (ppm):** 8.23 (m, 1H); 7.80 (m, 1H); 7.51 (m, 2H); 7.37 (m, 3H); 4.87 (s, 2H); 3.89 (s, 3H); 3.16 (s, 3H); 2.22 (s, 3H).**^13^C NMR (100 MHz, DMSO 298 K) (ppm):** 166.72, 166.30, 161.44, 151.07, 146.88, 134.47, 132.92, 132.37, 130.41, 129.39, 129.26, 127.16, 125.47, 124.50, 123.18, 108.11, 52.34, 50.31, 35.86, 12.08. **MS** (ESI) *m/z***:** 377.9 [M + H], 399.8 [M + Na]

### Synthesis compound 3


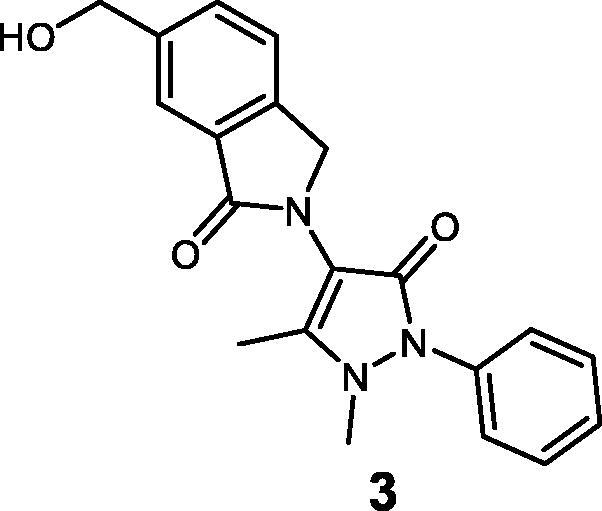


Borane dimethyl sulphide 1 M (0.165 ml, 0.165 mmol, and 0.6 eq) was added dropwise to a solution at 0 °C of compound **1** (100 mg, 0.275 mmol, and 1 eq) in dry DMSO. The mixture was stirred overnight at r.t. and then quenched with K_2_CO_3_. The solution was extracted with DCM (15 ml × 3). The combined organic layer wasw washed with HCl 1 N, brine, dried over Na_2_SO_4_ and concentrated.

**Yield:** quantitative

**^1^H NMR (400 MHz, DMSO, 298 K) (ppm):** 7.74 (s, 1H); 7.59 (m, 2H); 7.52 (m, 2H); 7.38 (m, 3H); 5.42 (s, OH); 4.77 (s, 2H); 4.63(s, 2H); 3.16 (s, 3H); 2.22 (s, 3H).**^13^C NMR (100 MHz, DMSO, 298 K) (ppm):** 167.42, 161.73, 153.18, 143.38, 141.43, 135.14, 131.83, 130.67, 129.65, 127.16, 124.53, 123.71, 121.35, 107.53, 62.98, 50.72, 36.09, 11.67. **MS** (ESI) *m/z***:** 371.9 [M + Na]

### Chemical stability of 1

^1^H NMR spectra were recorded after dilution of 10 mg of compound **1** in a mixture of DMSO/D_2_O 70:30. The chemical shift variation of the aromatic protons was used to monitor the behaviour of **1** in solution. NMR spectra were recorded after 2 h and 24 h. The chemical identity of compound **1** was confirmed by mass spectrometry after 24 h in solution (MS (ESI) *m/z*: 363.8 [M + H], 385.8 [M + Na]).

### Cell viability assay

As in a previous exercise, *in vitro* experiments on 14-3-3 PPI inhibitors were carried out using human erythroleukemia cell line K-562^17^. Cancer cells were cultured in RPMI medium with 10% FCS. In order to determine antiproliferative effect of compounds, K-562 cells were seeded at density of 5 × 10^4^ cells/ml, and treated with increasing concentrations of selected compounds. Control cells were treated with the vehicle of the experimental point containing the highest percentage of DMSO. Cell cultures were maintained at 37 °C in 5% v/v CO_2_ for 72 h. Cell number and vitality were evaluated on cell suspension using the automatic cell counter NucleoCounter® (Chemometec, Liller⊘d, Denmark). Each experiment was performed at least three times and results were expressed as mean and standard deviation (±SD).

### NMR spectroscopy

NMR spectra were performed at 14.1 T with a Bruker Avance 600 Spectrometer (Bruker) operating at controlled temperature (± 0.2 K) and using a 5 mm SEI probe. Chemical shifts were referenced to external 3-(Trimethylsilyl)propionic-2,2,3,3-d4 acid sodium salt (TMSP-d4). NOESY spectra were obtained by using standard pulse sequences. NOESY spectra were acquired with a mixing time of 320 ms.

### Molecular modelling

The crystallographic structure of 14-3-3σ in complex with a phosphopeptide coded by PDB ID: 1YWT was used as rigid receptor in molecular docking simulations, upon removal of the coordinates of the phosphopeptide and crystallographic water molecules^18^. Docking was carried out by the GOLD docking program version 5.0.1 (The Cambridge Crystallographic Data Centre, Cambridge, UK)^19^^,^^20^ using settings described previously^10^^,^^11^.

### Cells, cell culture, and transfections

HeLa cells stably expressing EGFP-Abl (HeLa EGFP-Abl) were obtained by transfection of HeLa cells with the pCEFL EGFP ABL wild-type expression vector^21^, using lipofectamine LTX (Life Technologies, Carlsbad, CA), according to the manufacturer’s instructions, followed by selection with 2 mg ml^− 1^ G418 (Sigma-Aldrich, St. Louis, MO) for 3 weeks. HeLa EGFP-Abl were maintained in DMEM supplemented with 10% FBS, 2 mM L-glutamine, 10,000 Uml^−1^ penicillin, and 10 mg ml^−1^ streptomycin and were constantly kept under selective pressure with 2 mgml^−1^ G418. Selective medium was replaced with regular growth medium on the day before experiments.

### Immunofluorescence, confocal microscopy, and intensitometric analysis of fluorescence

Cells were washed with PBS, then fixed with 4% paraformaldehyde in PBS for 20 min and permeabilised with 0.1% Triton X-100 in PBS for 30 min. Nuclei were stained with a solution of 6 μm of 4′,6-diamidino-2-phenylindole (DAPI; Sigma-Aldrich) in PBS for 10 min. Coverslips were mounted in fluorescence mounting medium (Dako, Agilent Technologies). Samples were visualised on a TSC SP5 confocal microscope (Leica, 5100000750), installed on an inverted LEICA DMI 6000CS (10741320) microscope and equipped with an oil immersion PlanApo 40 × 1.25 NA objective. Images were acquired using LAS AF acquisition software (Leica Camera, Wetzlar, Germany). Intensitometric analysis of fluorescence was performed using the Quantitation Module of the Volocity software (PerkinElmer Life Science). Briefly, total nuclear EGFP fluorescence, defined as EGFP signal co-staining with DAPI nuclear dye, was measured in seven representative confocal fields for each experimental condition. The resulting mean values ± SEM are expressed as a percentage of nuclear EGFP fluorescence. Seven representative fields were acquired and analysed for each sample. Significance (*p* values) was assessed by *t*-test. Asterisks were attributed for the following significance values: ^*****^p < .0001.

### Protein expression and purification

The gene encoding for human 14-3-3σ (h14–3-3σ) cloned in the pPROEX-HTb vector (including a TEV-cleavable His^6^-tag) between the BamHI and NotI restriction site, was a kind gift from Prof. C. Ottmann (Research group of Chemical Biology, Department of Biomedical Engineering, Eindhoven University of Technology, Netherlands). The expression plasmid was introduced by thermal shock in the *Escherichia coli* strain BL21(DE3). Transformants were selected on LB-agar plates added by 100 mgl^−1^ ampicillin. Bacterial cells were cultured in LB medium, supplemented with 100 mgl^−1^ ampicillin at 37 °C under vigorous aeration. When OD_600nm_ reached values ranging from 0.6 to 0.8, protein over-expression was induced by adding 0.5 mM IPTG to the culture that was then incubated at 25 °C for 16 h (under vigorous aeration). Cells, harvested by centrifugation, were resuspended in buffer A (250 mM NaCl and 50 mM Tris-HCl, pH 8) added by 20 mM imidazole and lysozyme (0.5 mg ml^−1^) and disrupted by sonication after 1 h of incubation on ice. The soluble cellular fraction, clarified by centrifugation (13500 × *g*, 1 h, 4 °C), was purified by taking advantage of the N-terminal His^6^-tag using nickel affinity chromatography (HisTrap FF 5 ml column, GE Healthcare, Chicago, IL). The purification protocol relied on a three-step gradient imidazole concentration (in the same buffer) that showed elution of the His^6^-tag h14-3-3σ at imidazole concentrations ranging from 75 to 250 mM. Fractions containing the target protein (identified by SDS-PAGE) were pooled and dialysed in 3 mM Tris-HCl, pH 8 at 4 °C. The final protein yield resulted of 87 mg l^−1^ bacterial culture.
